# Antibiotic Prescribing Patterns and Antimicrobial Stewardship Among Indian Periodontists: A Cross-Sectional Survey

**DOI:** 10.3390/healthcare14193226

**Published:** 2026-10-01

**Authors:** Ankita Chhabrani, Giuseppe D’Albis, Avinash Bettahalli Shivamallu, Saverio Capodiferro

**Affiliations:** 1 Department of Periodontology, Manav Rachna Dental College, Manav Rachna International Institute of Research and Studies, Faridabad 121004, Haryana, India; 2 Department of Interdisciplinary Medicine, University of Bari Aldo Moro, 70121 Bari, Italy; saverio.capodiferro@uniba.it; 3 Department of Periodontology, JSS Dental College and Hospital, JSS Academy of Higher Education and Research, Mysuru 570015, Karnataka, India; dr.avinashbs@jssuni.edu.in

**Keywords:** antibiotics, resistance, public health, periodontology, global concern, antibiotic stewardship

## Abstract

**Background**: Antimicrobial resistance is a growing concern, and inappropriate prescribing may contribute to its rise. Variability among periodontists highlights the need to understand prescribing patterns and factors influencing antibiotic selection. We aim to assess antibiotic prescribing patterns and perceptions of resistance among Indian periodontists and evaluate factors associated with prescribing practices and selection of the antibiotic regimen perceived by respondents as most effective. **Methods**: A cross-sectional questionnaire survey was conducted among Indian periodontists (January–April 2024), distributed via institutional email and LinkedIn. Descriptive statistics summarized responses; chi-square/Fisher’s tests assessed associations with experience. Logistic regression was performed to identify factors associated with respondents’ selection of the antibiotic regimen perceived as most effective for periodontal therapy. **Results**: Of 500 invited, 328 responded (65.6%). Respondents were mostly female (67.7%), with a mean age of 32.25 ± 6.05 years; 50.3% had <5 years’ experience and 98.6% held an MDS in Periodontology. Amoxicillin plus metronidazole was the regimen most frequently perceived as effective by respondents (37.8%), followed by amoxicillin–clavulanic acid (35.4%) and amoxicillin alone (15.9%). Oral administration was preferred (89.0%). Prophylactic antibiotics were prescribed for infective endocarditis/CABG indications by 89.6%, while 48.2% did not prescribe after laser-assisted surgery. Experience was significantly associated with prescribing frequency and post-laser prescribing (*p* < 0.05); associations with crown lengthening and flap surgery lost significance after Bonferroni correction. Academic practice was associated with regimen selection univariably (OR = 0.56, 95% CI 0.34–0.93) but not after multivariable adjustment (aOR = 0.65, 95% CI 0.37–1.13). Most respondents (98.5%) viewed resistance as a growing concern. **Conclusions**: Prescribing practices varied with experience and practice setting. Amoxicillin plus metronidazole was most frequently considered effective, though no factor independently predicted selection. High awareness of resistance underscores the need for stronger antimicrobial stewardship in periodontal care.

## 1. Introduction

The World Health Organization (WHO) recognizes antimicrobial resistance (AMR) as a major global health concern, driven by factors including widespread antimicrobial use in agriculture, inappropriate prescribing by healthcare providers, and self-medication, resulting in increased morbidity, mortality, and healthcare expenditure [1]. Inappropriate antibiotic use in dental practice represents an important and potentially modifiable contributor to antimicrobial resistance. Within periodontal care, judicious antibiotic prescribing is therefore essential to ensure appropriate clinical management while minimizing unnecessary antimicrobial exposure.

Periodontal infections such as peri-implantitis and periodontitis may require systemic antibiotics as an adjunct to mechanical periodontal therapy in selected clinical situations [2]. However, the lack of standardized guidelines for antibiotic prescription in periodontal diseases contributes to considerable variability in prescribing behaviours among practitioners [3]. Despite existing recommendations advocating cautious and evidence-based antibiotic use, India, like many other regions, lacks specific standardized clinical practice guidance addressing antibiotic prescribing in periodontal practice. This may contribute to differences in antibiotic selection and prescribing practices across clinicians and practice settings.

Given the global urgency surrounding antimicrobial resistance and the variability in antibiotic prescribing practices, there is a need to characterize antibiotic prescribing practices among Indian periodontists and to identify professional factors associated with such practices. Examining differences according to years of clinical experience and practice setting may provide insight into factors contributing to prescribing variability and help identify areas for targeted antimicrobial stewardship. To date, there is limited evidence describing antibiotic prescribing practices and perceptions regarding antimicrobial resistance among periodontists in India.

Therefore, this study aimed to assess antibiotic prescribing patterns and attitudes toward antimicrobial resistance among Indian periodontists, including antibiotic selection, prescribing frequency, routes of administration, and clinical and prophylactic indications. The study further evaluated whether prescribing practices differed according to years of clinical experience and practice setting and identified professional factors independently associated with selection of the antibiotic regimen considered most effective for periodontal therapy.

## 2. Materials and Methods

### 2.1. Study Design, Setting and Participants

The study was designed as a cross-sectional, questionnaire-based survey targeting qualified periodontists in India. The study was conducted between January and April 2024. The study population comprised dental graduates who had completed postgraduate specialization in periodontology (MDS or an equivalent recognized postgraduate qualification) and were practicing as periodontists in India. BDS graduates without postgraduate specialization in periodontology were excluded from the study. A total of 500 periodontists were approached, of whom 328 completed the questionnaire, yielding a response rate of 65.6%. Participants were recruited through LinkedIn and institutional email by approaching periodontists practicing in different geographical regions of India, including North, South, East, West, Central and Northeast India. A follow-up reminder was sent after one week. A non-probability convenience sampling approach was used; therefore, the sample should not be considered nationally representative of all practicing periodontists in India. The questionnaire was administered using Google Forms, with the “Limit to one response” option. Although the questionnaire included a name field, names were collected solely for participant identification and data-validation purposes, including identification and removal of duplicate responses. Names were not used for individual-level analysis or reporting. Following completion of data validation and duplicate checking, identifying information was removed from the dataset used for statistical analysis. The participant information was handled confidentially and the final analytical dataset was de-identified. All findings were reported in aggregate form without disclosure of individual participant identities.

### 2.2. Sample Size

The sample size was calculated using the formula:

$$S=\frac{X^{2}\mathrm{NP}\left(1-P\right)}{d^{2}\left(N-1\right)+X^{2}P\left(1-P\right)}$$

where X^2^ = 3.841, N = 3000 (based on the data found on the official website of the Indian Society of Periodontology), P = population proportion (0.5), and d = degree of accuracy (0.05). Based on this calculation, the minimum required sample size was estimated to be 341 participants. Expecting 30% attrition, the total required sample was 488. Therefore 500 periodontists were approached.

### 2.3. Questionnaire Design and Validation

The survey was developed to investigate antibiotic prescribing practices among Indian periodontists, including antibiotic selection, prescribing frequency, routes of administration, prescribing practices in specific clinical situations, and perceptions regarding antimicrobial resistance. An online questionnaire was developed based on existing questionnaires reported in the literature [4,5].

The questionnaire consisted of three sections comprising 17 questions, with a combination of single-response and multiple-response items. The first section collected general and professional characteristics, including sociodemographic and educational information. The second section assessed antibiotic prescribing practices, including antibiotic selection and prescribing practices in different clinical and prophylactic situations. This section also included a question assessing which antibiotic regimen respondents considered most effective for periodontal therapy. The third section assessed attitudes and perceptions regarding antimicrobial resistance.

The questionnaire comprised heterogeneous items covering different domains, including antibiotic prescribing practices, clinical and prophylactic indications, and perceptions regarding antimicrobial resistance. These items were intended to assess distinct aspects of antibiotic prescribing behavior and perceptions rather than a single underlying construct. Cronbach’s alpha was initially 0.35 and was recalculated following refinement of the questionnaire, yielding a coefficient of 0.40. Although the alpha value remained low, it was interpreted in the context of the heterogeneous nature of the questionnaire and the absence of a single underlying construct across all items. Therefore, Cronbach’s alpha was not considered an appropriate measure of the internal consistency of the final questionnaire. The questionnaire was retained based on its content relevance, face validity, and comprehensive coverage of the predefined domains of the study.

To assess the questionnaire’s clarity, relevance, and comprehensiveness, 10 periodontists who were not involved in the main study were selected to review the questionnaire during its preliminary development phase. They assessed the face and construct validity of the questionnaire. Following this preliminary evaluation and refinement, the final questionnaire was distributed anonymously to periodontists across India. The study was reported in accordance with the Strengthening the Reporting of Observational Studies in Epidemiology (STROBE) guidelines for cross-sectional studies [6]. The completed STROBE checklist is provided as Supplementary Materials.

### 2.4. Statistical Analyses

The collected responses were analyzed using IBM SPSS Statistics version 23.0 (IBM Corp., Chicago, IL, USA). Descriptive statistics were used to summarize responders’ characteristics and questionnaire responses, and data were presented as frequencies and percentages. Chi-square or Fisher’s exact tests were performed to find associations between questionnaire response variables and years of clinical experience.

Univariate and multivariate binary logistic regression analysis were performed to identify professional factors independently associated with respondents’ selection of amoxicillin + metronidazole as the regimen perceived to be most effective for periodontal therapy. The selection of this regimen was based on respondents’ reported perceptions and was not intended to imply that it is universally recommended for routine periodontal use. The factors considered were gender, clinical experience, practice type, and post-operative condition. The variables having a *p*-value < 0.30 in univariable analysis were considered in multivariate logistic regression. Unadjusted and adjusted odds ratios (ORs) with corresponding 95% confidence intervals (CIs) were reported.

A two-sided *p*-value of <0.05 was considered statistically significant.

The antibiotics reported by respondents were classified according to the World Health Organization (WHO) AWaRe (Access, Watch and Reserve) classification. The WHO AWaRe classification was used to categorize the individual antibiotics reported in response to the questionnaire item assessing the antibiotic regimen perceived as most effective by respondents. Each respondent contributed one response to this item. Frequencies and percentages were calculated using the total number of valid responses (n = 328) as the denominator. Antibiotic combination regimens identified by WHO as Not Recommended were analysed separately and were not assigned to an individual AWaRe category.

## 3. Results

A total of 328 of the 500 invited periodontists responded to the survey, yielding a response rate of 65.6%. Among the respondents, 134 (40.8%) were aged 25–30 years, 100 (30.5%) were aged 30–35 years, 60 (18.3%) were aged 35–40 years, and 34 (10.4%) were aged ≥40 years. The mean age of the respondents was 32.25 ± 6.05 years (range: 25–69 years). Female respondents constituted 222 (67.7%) of the sample, while 106 (32.3%) were male. The mean age of male respondents was higher than that of female respondents (34.57 ± 7.93 vs. 31.30 ± 4.56 years), with a mean difference of 3.27 years (*p* < 0.001).

Regarding clinical experience, 165 (50.3%) respondents had <5 years of clinical practice, 91 (27.7%) had 5–9 years, 40 (12.2%) had 10–14 years, and 32 (9.8%) had ≥15 years of clinical experience. The median duration of clinical experience was 4.0 years, ranging from 1 to 40 years. Most respondents, 320 (98.6%), had an MDS qualification in Periodontology, while 6 (1.8%) held a PhD and 2 (0.6%) were pursuing a PhD, depicted in Table 1.

Private practice was the most frequently reported practice setting (242/328, 73.8%), followed by academic institutions (98/328, 29.9%), hospital-based practice (54/328, 16.5%), and healthcare/trust settings (24/328, 7.3%). As respondents were permitted to report more than one practice setting, the percentages do not sum to 100%.

Amoxicillin in combination with metronidazole was the antibiotic regimen most frequently perceived as effective by respondents, selected by 124 (37.8%) participants, followed by amoxicillin–clavulanic acid in 116 (35.4%) and amoxicillin alone in 52 (15.9%).

The remaining respondents selected other antibiotic regimens, each of which was reported by relatively few participants. Oral administration was preferred by 292 (89.0%) respondents, whereas 36 (11.0%) preferred local drug delivery. For pregnant patients, 170 (51.8%) respondents reported preferring amoxicillin, while 120 (36.6%) preferred referrals to a gynecologist. Following laser-assisted periodontal surgery, 158 (48.2%) respondents reported that they did not prescribe antibiotics.

Bone grafting was the most frequently reported postoperative condition associated with antibiotic prescription, followed by implant-related procedures and abscess drainage. A total of 294 (89.6%) respondents reported prescribing antibiotics for patients with infective endocarditis, coronary artery bypass grafting (CABG), or related indications. As these clinically distinct indications were assessed within a single question, the responses could not be retrospectively disaggregated to determine the proportion of respondents prescribing antibiotics specifically for infective endocarditis versus CABG-related indications as a prophylactic measure. Almost all respondents, 323 (98.5%), considered antibiotic resistance to be a growing concern.

A statistically significant association was observed between clinical experience and the frequency of antibiotic prescription for periodontal infections (*p* = 0.024). Clinical experience was also significantly associated with antibiotic prescription following laser-assisted periodontal surgery (*p* = 0.048), as well as antibiotic prescription following crown lengthening (*p* = 0.008) and flap surgery (*p* = 0.009). However, following Bonferroni adjustment for the multiple comparisons, the associations observed for crown lengthening and flap surgery were no longer statistically significant. Other prescribing characteristics did not demonstrate statistically significant associations with clinical experience. Because multiple responses were permitted for practice setting, factors influencing brand selection, and postoperative conditions, these variables were analyzed descriptively where appropriate and percentages were not expected to sum to 100% as depicted in Table 2.

### 3.1. Descriptive Comparison of Antibiotic Prescribing Practices Across Practice Settings

Private practice was the most frequently reported practice setting. Among respondents reporting hospital-based practice, 24/54 (44.4%) reported prescribing antibiotics either hardly ever or never, compared with 20.4% of respondents reporting academic institutional practice, 25.0% reporting healthcare/trust practice, and 23.9% reporting private practice. The distribution of antibiotic prescribing frequency, antibiotic selection, factors influencing brand selection, prescribing for infective endocarditis/CABG-related indications, antibiotic use following laser-assisted periodontal surgery, and postoperative conditions across practice settings is presented in Table 3. Because respondents could select more than one practice setting and more than one postoperative condition, the totals and percentages for these variables may exceed the total study population and 100%, respectively.

### 3.2. Factors Associated with Selection of the Antibiotic Regimen Perceived as Most Effective by Respondents

Univariable and multivariable binary logistic regression analyses were performed to identify factors associated with selection of amoxicillin plus metronidazole as the antibiotic regimen considered most effective by respondents. In the univariable analysis, reporting practice in an academic institution was significantly associated with selection of amoxicillin plus metronidazole (OR = 0.56, 95% CI: 0.34–0.93; *p* = 0.023). Clinical experience, private practice, hospital-based practice, healthcare/trust practice, and the assessed postoperative conditions were not statistically significantly associated with the selection of amoxicillin plus metronidazole in the univariable analysis, depicted in Table 4.

The overall classification of the model = 62.5%, with Negaelkerke R-square 0.081. Variables with *p* < 0.30 in the univariable analysis were considered for inclusion in the multivariable logistic regression model. These included academic institution, private practice, bone grafting, flap surgery, crown lengthening, and gingivectomy. Scaling was not included in the multivariable model because of the small number of observations.

After multivariable adjustment, the association between academic institutional practice and selection of amoxicillin plus metronidazole was no longer statistically significant (adjusted OR = 0.65, 95% CI: 0.37–1.13; *p* = 0.130). None of the other variables included in the multivariable model demonstrated a statistically significant independent association with selection of amoxicillin plus metronidazole. The overall classification accuracy of the model was 62.5%, with a Nagelkerke R^2^ of 0.081.

**Figure 1:**
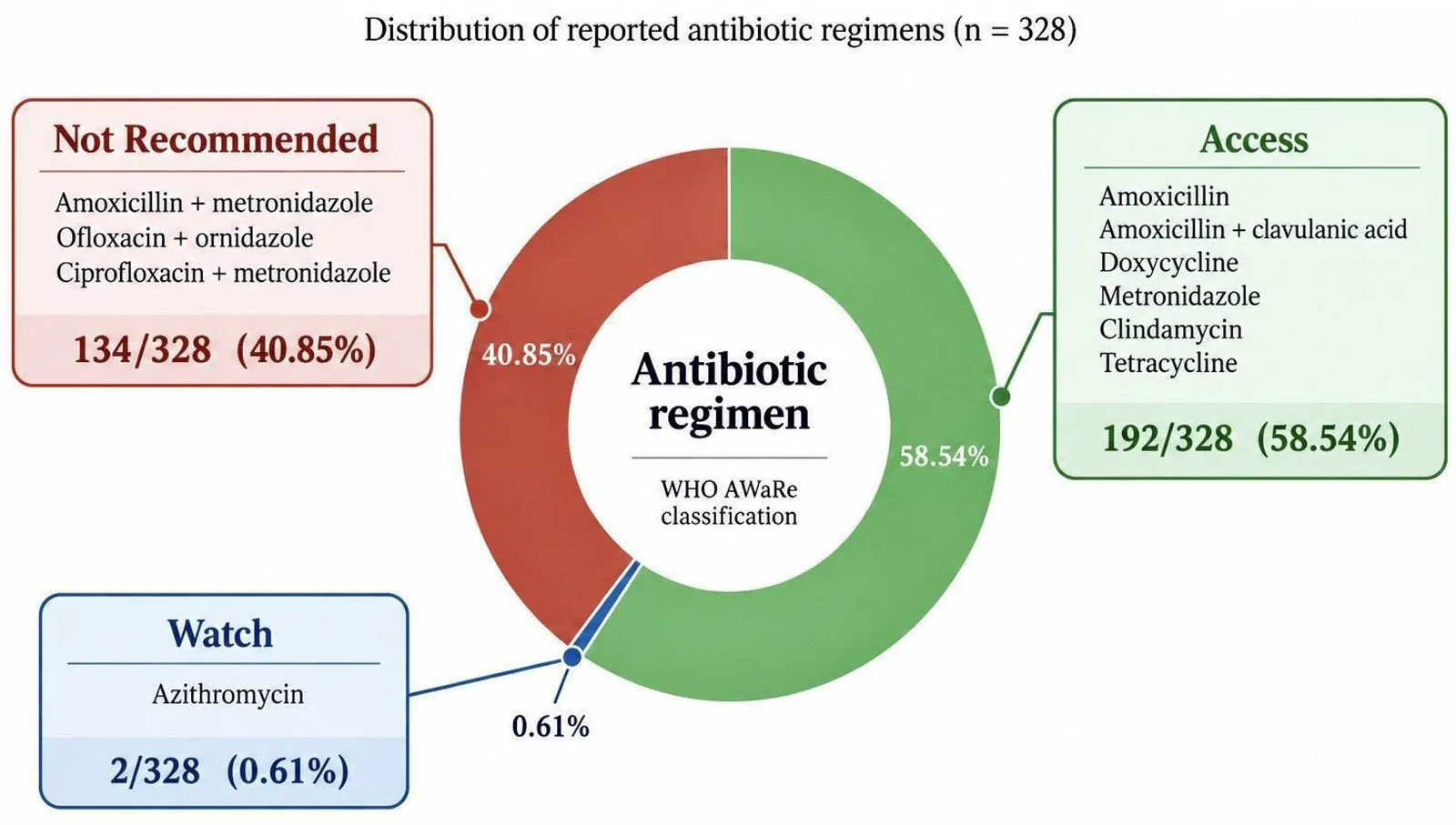
The WHO AWaRe classification with categorization of various antibiotics. Adapted from [7] and redrawn using biorender.

The antibiotics reported by respondents were classified according to the WHO AWaRe classification. Of the 328 reported regimens, 58.54% were categorized as Access, 0.61% as Watch, and 40.85% as “Not Recommended” combinations (Figure 1).

## 4. Discussion

The present study identified substantial variation in antibiotic prescribing among Indian periodontists by clinical experience and practice setting. Academic institutional practice was associated with selecting amoxicillin plus metronidazole univariably, but not after multivariable adjustment. Prescribing frequency for periodontal infections and after laser-assisted surgery differed significantly by experience; associations with crown lengthening and flap surgery lost significance after Bonferroni correction. Similar variation has been reported previously [4,5], including in Belgium, despite good resistance awareness [6,7,8]. In total, 65.9% of respondents often prescribed antibiotics for periodontal infections, 23.2% hardly ever, and 0.6% never, higher than reported in Germany [5], possibly reflecting differences in recommendations, systems and training. Evidence from India similarly highlights prescribing heterogeneity and the need for stronger stewardship [9]. Current evidence does not support routine systemic antibiotics for localized periodontal infections [10,11,12], though they may be warranted in severe or specific odontogenic infections [13,14,15]. Amoxicillin plus metronidazole was most frequently selected (37.8%), followed by amoxicillin–clavulanic acid (35.4%) and amoxicillin alone (15.9%), consistent with evidence supporting this combination in selected conditions [16,17], though perceived effectiveness should not be equated with an indication for routine use.

Penicillin-based prescribing predominates in the United States [18,19], while Australian periodontists prefer azithromycin with lower overall use. Reported agents are consistent with the broader literature on tetracyclines, metronidazole and penicillins for odontogenic infections [20]. Amoxicillin and amoxicillin–clavulanic acid remain common in dental practice [21,22], with continued amoxicillin use during implant procedures in Europe [23], greater clindamycin use in Germany [24], and variation among Singaporean specialists [25]. Practice-setting differences were apparent: 12.4% of private practitioners always prescribed for periodontal infections, versus 6.1–8.3% in academic, healthcare/trust and hospital settings; amoxicillin–clavulanic acid predominated in academic and hospital settings, and amoxicillin plus metronidazole in private practice [23,24,25,26]. Postoperative prescribing was common for bone grafting, abscess drainage and implant-related procedures [27], though routine antibiotics after uncomplicated procedures offer limited benefit and should be reserved for selected patients [28,29,30]. Amoxicillin–clavulanic acid’s added protection over alternatives has also been questioned [28]. Evidence on non-surgical therapy remains mixed [31,32,33].

Only 11% used locally delivered antibiotics, lower than in some European settings [4,5]. Antibiotics were frequently prescribed for bone grafting (78.0%) and implant procedures (73.8%), consistent with international patterns [23,24], though evidence suggests infection rates may not differ substantially with routine use, supporting a more selective approach [34]; evidence on peri-implantitis remains heterogeneous [35]. High resistance awareness (98.5%, consistent with Australian periodontists) coexisted with substantial prescribing variability, indicating awareness alone does not ensure evidence-based prescribing [36]. In logistic regression, academic practice was associated with selecting amoxicillin plus metronidazole univariably (OR = 0.56, 95% CI 0.34–0.93; *p* = 0.023) but not after adjustment (aOR = 0.65, 95% CI 0.37–1.13; *p* = 0.130); the low Nagelkerke R^2^ (0.081) indicates prescribing is multifactorial. Limitations include the cross-sectional design, self-reported prescribing subject to bias, possible selection/non-response bias from recruitment via email and LinkedIn, multiple-response items complicating comparisons, and a low Cronbach’s alpha (α = 0.40). No independent predictor of amoxicillin–metronidazole selection emerged after adjustment. High resistance-related concern combined with prescribing variability underscores the need for evidence-based, context-specific stewardship, supported by standardized guidance, continuing education and further research [37,38].

### Antibiotic Stewardship in Periodontal Practice: Translating Survey Findings into Clinical Action

Antibiotic stewardship is essential for combating antimicrobial resistance in dentistry. Inappropriate prescribing, driven by diagnostic uncertainty, patient demand, inadequate knowledge, and time constraints, remains the primary driver of rising resistance [39,40]. Although 98.5% of respondents recognized antimicrobial resistance as a growing concern, this awareness did not translate into homogeneous prescribing behaviour. Clinical experience was significantly associated with prescribing frequency for periodontal infections (*p* = 0.024), with respondents having ≥15 years of experience more likely to report “always” prescribing (25.0%) than those with <5 years (8.5%). Significant experience-related differences also emerged for prescribing after laser-assisted surgery (*p* = 0.048), crown lengthening (*p* = 0.008), and flap surgery (*p* = 0.009), though the latter two lost significance after Bonferroni correction. A similar pattern was seen across practice settings, with hospital-based respondents most often prescribing “hardly ever/never” (44.4%) compared with private (23.9%), healthcare/trust (25.0%), and academic settings (20.4%). These findings indicate that awareness alone does not ensure uniform, evidence-based prescribing, underscoring the need for standardized guidelines and continuing education [37,38].

Stewardship requires prudent antibiotic use guided by accurate diagnosis, with prescribing reserved for clinically indicated cases and appropriate selection, dosage, route and duration [39,41]. In our sample, this was applied selectively: 89.6% prescribed for infective endocarditis/CABG, and prescribing clustered around bone grafting, implants, and abscess drainage, procedures with recognized infective risk. For pregnant patients, 51.8% preferred amoxicillin versus 36.6% preferring gynaecological referral. Despite this apparent risk-stratification, logistic regression found no independent association between postoperative conditions or clinical experience and selection of the perceived most-effective regimen; academic practice was associated with reduced odds of selecting amoxicillin plus metronidazole univariably (OR = 0.56, 95% CI 0.34–0.93; *p* = 0.023) but not after adjustment (aOR = 0.65, 95% CI 0.37–1.13; *p* = 0.130). The low Nagelkerke R^2^ (0.081) suggests regimen selection is shaped by unmeasured factors such as training background or local case mix rather than standardized protocols.

Amoxicillin plus metronidazole was the regimen most frequently perceived by respondents as effective (37.8%). Notably, this combination was classified as “Not Recommended” within the WHO AWaRe framework. This apparent discrepancy reflects the different purposes of the two perspectives: periodontal evidence evaluates the clinical efficacy of adjunctive antimicrobial therapy in selected patients, whereas the WHO AWaRe framework is primarily intended to support antimicrobial stewardship and appropriate antibiotic use. Therefore, the “Not Recommended” classification should not be interpreted as indicating a lack of periodontal clinical efficacy of amoxicillin plus metronidazole. Rather, the finding highlights a potential gap between perceived clinical effectiveness and antimicrobial-stewardship recommendations.

In the present survey, the AWaRe classification revealed that a substantial proportion of reported antibiotic regimens fell within the “Not Recommended” category, largely reflecting combination regimens such as amoxicillin plus metronidazole. This finding illustrates that a regimen may be perceived by clinicians as clinically effective in selected periodontal conditions while still requiring cautious and selective use from an antimicrobial-stewardship perspective [7]. Previous periodontal evidence supporting the adjunctive use of amoxicillin plus metronidazole in selected clinical situations may provide context for its frequent selection by respondents; however, perceived effectiveness should not be equated with an indication for routine prescribing. Antibiotic selection should therefore balance demonstrated clinical efficacy with antimicrobial-resistance risk and stewardship principles. Access antibiotics are generally preferred as first-line agents because of their more favourable resistance profiles, whereas Watch antibiotics warrant greater caution and Reserve antibiotics should be restricted to situations in which their use is specifically justified [7].

Patient education on the limited indications for antibiotics after routine periodontal therapy, relevant given that 65.9% of respondents “often” prescribed for periodontal infections, may reduce prescribing pressure [36]. The experience- and setting-related differences observed suggest that continuing education should be tailored to experience level and practice context rather than delivered uniformly [42].

Addressing antimicrobial resistance requires coordinated efforts beyond prescribing alone: infection prevention, evidence-based guidelines, vaccination, environmental control, surveillance, public education, and research into novel therapeutics [7,37].

Given that high awareness coexisted with measurable prescribing variability in this survey, isolated awareness campaigns are unlikely to suffice; stewardship must combine standardized protocols, targeted education, and institutional surveillance to close the gap between recognizing resistance as a concern and consistently achieving AWaRe-concordant prescribing.

## 5. Conclusions

The present study demonstrated variation in reported antibiotic prescribing practices among Indian periodontists, with some prescribing responses differing across clinical-experience groups and practice settings, despite a high level of awareness regarding antimicrobial resistance. Amoxicillin plus metronidazole was the regimen most frequently considered effective by respondents; however, no independent professional or clinical predictors of its selection were identified after multivariable adjustment. These findings underscore the need for standardized, evidence-based guidance for antibiotic prescribing in periodontal practice, with consideration of clinical indications, antimicrobial resistance patterns, and appropriate antibiotic selection, dosage, route, and duration. Strengthening antimicrobial-stewardship education and incorporating practical prescribing guidance into continuing professional development may help reduce unwarranted variation in antibiotic use and support the rational use of antimicrobials in periodontal care.

## Figures and Tables

**Table 1:** Descriptive statistics of the responders.

		Numbers (Percentage)
Age of Responders (Years)	25–30	134/328 (40.8)
	30–35	100/328 (30.5)
	35–40	60/328 (18.3)
	>=40	34/328 (10.4)
Gender	Male	106/328 (32.3)
	Female	222/328 (67.7)
Education Qualification	MDS	320/328 (98.6)
	PhD	6/328 (1.8)
	Ongoing PhD Scholar	2/328 (0.6)
Number of years of clinical practice	<5 years	165/328 (50.3)
	5–9 years	91/328 (27.7)
	10–14 years	40/328 (12.2)
	>15 years	32/328 (9.8)
Practice type *	Academic Institution	98/328 (29.9)
	Health care/trust	24/328 (7.3)
	Hospital based	54/328 (16.5)
	Private	242/328 (73.8)

* Total percentage is more than 100 due multiple response.

**Table 2:** Association of experience in clinical practice (number of years) with questionnaire.

Question		Experience in Clinical Practice (Number of Years)				*p* Value
		<5 (n = 165)	5–9 (n = 91)	10–14 (n = 40)	>15 (n = 32)	
		%	%	%	%	
How often do you prescribe antibiotics for periodontal infections?	Always (n = 34)	14(8.5)	10(10.9)	2(5.0)	8(25.0)	**0.024 #** **(Fisher’s Exact)**
	Hardly ever (n = 76)	42(24.4)	20(22.0)	8(20.0)	6(18.8)	
	Never (n = 2)	0(0.0)	0(0.0)	0(0.0)	2(6.2)	
	Often (n = 216)	109(66.1)	61(67.0)	30(75.0)	16(50.0)	
Which among the following factors is your prime consideration while prescribing a particular brand of antibiotics? *	Availability of the brand [n = 194]	93(56.4)	51(56.0)	30(75.0)	20(62.5)	0.158
	Popularity of the brand [n = 74]	40(24.2)	20(22.0)	10(25.0)	4(12.5)	0.517
	Affordability of brand [n = 155]	88(53.3)	37(40.7)	16(40.0)	14(43.8)	0.169
Which is the most effective antibiotic prescribed by you?	Amoxicillin [n = 52]	36(21.8)	10(10.9)	2(5.0)	4(12.5)	**Chi-square = 27.71,** ***p* = 0.006**
	Amoxicillin + Metronidazole [n = 124]	68(41.2)	30(32.6)	16(40.0)	10(31.2)	
	Amoxicillin-clavulanic acid [n = 116]	49(29.7)	43(47.8)	12(30.0)	12(37.5)	
	Doxycycline [n = 14]	6(3.6)	2(2.2)	4(10.0)	2(6.2)	
	Others $	6(3.6)	6(6.6)	6(15.0)	4(12.4	
	Azithromycin *$ [n = 2]	**2(1.2)**	**0(0.0)**	**0(0.0)**	**0(0.0)**	
	**Ciprofloxacin metronidazole $ [n = 2]**	**0(0.0)**	**2(2.2)**	**0(0.0)**	**0(0.0)**	
	**Clindamycin $ [n = 2]**	**0(0.0)**	**0(0.0)**	**0(0.0)**	**2(6.2)**	
	Metronidazole $* [n = 6]	**0(0.0)**	**4(4.4)**	**2(5.0)**	**0(0.0)**	
	**Ofloxacin + Ornidazole $ [n = 8]**	**4(2.4)**	**0(0.0)**	**4(10.0)**	**0(0.0)**	
	**Tetracycline $ [n = 2]**	**0(0.0)**	**0(0.0)**	**0(0.0)**	**2(6.2)**	
Do you prescribe antibiotics for infective endocarditis, CABG?	No [n = 34]	18(11.0	8(8.8)	4(10.0	4(12.5)	0.909
	Yes [ n = 294]	147(89.0)	83(91.2)	36(90.0)	28(87.5)	
Do you prescribe antibiotics after laser-assisted periodontal surgeries?	Maybe [n = 100]	42(24.4)	40(43.5)	10(25.0)	8(25.0)	**0.048** **(chi-square = 12.70)**
	No [n = 158]	85(52.4)	37(41.3)	22(55.0)	14(43.8)	
	Yes [n = 70]	38(23.2)	14(15.2)	8(20.0)	10(31.2)	
Post-operative condition for prescription *	Scaling [n = 6]	4(2.4)	0(0.0)	2(5.0)	0(0.0)	**0.158#**
	Abscess drainage [n = 253]	124(75.2)	71(78.0)	32(80.0)	26(81.2)	0.825
	Frenectomy [n = 40]	24(14.5)	10(11.0)	2(5.0)	4(12.5)	0.427
	Gingivectomy [n = 40]	22(13.4)	8(8.8)	6(15.0)	4(12.5)	0.657
	Crown lengthening [n = 46]	22(13.4)	6(6.6)	10(21.7)	8(25.0)	0.008
	Flap surgery [n = 193]	91(55.2)	56(61.5)	32(80.0)	14(43.8)	0.009
	Bone graft [n = 256]	129(78.2)	73(80.2)	30(75.0)	24(75.0)	0.887
	Implant [n = 242]	125(75.8)	63(69.2)	32(80.0)	22(68.8)	0.471
Do you believe that antibiotic resistance is of growing concern?	No [n = 5]	3(1.8)	2(2.2)	0(0.0)	0(0.0)	1.00#
	Yes [n = 323]	162(98.2)	89(97.8)	40(100.0)	32(100.0)	
First-choice antibiotic prescribed to patients allergic to Pencillin	Spirameyin + Metronidazole [n = 18]	8(4.8)	8(8.8)	0(0.0)	2(6.3)	Chi-square value = 59.996, *p* < 0.001
	Clindamycin [n = 94]	52(3.5)	24(26.4)	12(30.0)	6(18.8)	
	Clarithromycin [n = 30]	22(11.0)	8(8.8)	0(0.0)	0(0.0)	
	Azithromycin [n = 166]	77(46.7)	45(49.5)	26(65.0)	18(56.3)	
	Others (Specify) [n = 28]	6(3.64)	6(6.6)	2(5.0)	14(3.8)	
First choice of antibiotic for a pregnant patient	Spiramycin [n = 2]	2(1.2)	0(0.0)	0(0.0)	0(0.0)	Chi-square value = 18.738, *p* = 0.028
	Amoxicillin + Clavulantic acid [n = 36]	16(9.7)	8(8.8)	8(20.0)	4(12.5)	
	Amoxicillin [n = 170]	83(50.3)	51(56.0)	26(65.0)	10(31.2)	
	Refer to gynaecologist [n = 120]	64(38.8)	32(35.2)	0(0.0)	18(56.2)	

* Multiple responses were permitted for these items; therefore, category totals may exceed 328 and percentages may not sum to 100%. Each category was analysed separately; # Fisher’s exact test, $ collapsed in the other categories for finding association. $ Individual low-frequency antibiotic/antibiotic-combination responses were collapsed into the “Others” category for finding association.

**Table 3:** The table shows the association between practice type and questionnaire.

Question		Practice Type * [n = 328]			
	Total = 418 [Due to Multiple Responses]	Academic Institution [n = 98]	Health Care/ Trust [n = 24]	Hospital Based [n = 54]	Private [n = 242]
		%	%	%	%
How often do you prescribe antibiotics for periodontal infections?	Always	6(6.1)	2(8.3)	4(7.4)	30(12.4)
	Hardly ever	20(20.4)	6(25.0)	22(40.7)	56(23.1)
	Never	0(0.0)	0(0.0)	2(3.7)	2(0.8)
	Often	72(73.5)	16(66.7)	26(48.1)	154(63.6)
Which of the following factors do you consider primarily while prescribing a particular brand of antibiotics? *	Availability of the brand [n = 194]	54(55.1)	14(58.3)	28(51.9)	154(63.6)
	Popularity of the brand [n = 74]	18(18.4)	10(41.7)	18(33.3)	56(23.1)
	Affordability of brand [n = 155]	54(55.1)	14(58.3)	24(44.4)	109(45.0)
What is the most effective antibiotic prescribed by you?	Amoxicillin	22(22.4)	2(8.3)	6(11.1)	32(13.2)
	Amoxicillin + Metronidazole	28(28.6)	8(33.3)	20(37.0)	98(40.5)
	Amoxicillin–clavulanic acid	40(40.8)	10(41.7)	20(37.0)	84(34.7)
	Azithromycin	0(0.0)	0(0.0)	0(0.0)	2(0.8)
	Ciprofloxacin metronidazole	0(0.0)	0(0.0)	0(0.0)	2(0.8)
	Clindamycin	2(2.0)	0(0.0)	0(0.0)	2(0.8)
	Doxycycline	4(4.1)	0(0.00	6(11.1)	8(3.3)
	Metronidazole	2(2.0)	0(0.0)	0(0.0)	4(1.7)
	Ofloxacin + Ornidazole	0(0.0)	4(16.7)	2(3.7)	8(3.3)
	Tetracycline	0(0.0)	0(0.0)	0(0.0)	2(0.8)
	Spiramycin + Metronidazole	0(0.0)	0(0.0)	0(0.0)	0(0.0)
Do you prescribe antibiotics for infective endocarditis, CABG etc.?	No	12(12.2)	0(0.0)	6((11.1)	20(8.3)
	Yes	86(87.8)	24(100.0)	48(88.9)	222(91.7)
Do you prescribe antibiotics after laser-assisted periodontal surgeries?	Maybe	26(26.5)	4(16.7)	14(25.9)	78(32.2)
	No	50(51.0)	14(58.3)	26(48.1)	110(45.5)
	Yes	22(22.4)	6(25.0)	14(25.9)	54(22.3)
Post-operative conditions for prescription *	Scaling	2(2.0)	0(0.0)	2(3.7)	2(0.8)
	Abscess drainage	64(65.3)	14(58.3)	40(74.1)	181(74.8)
	Frenectomy	14(14.3)	2(8.3)	4(7.4)	26(10.7)
	Gingivectomy	10(10.2)	0(0.0)	2(3.7)	32(13.2)
	Crown lengthening	10(10.2)	0(0.0)	6(11.1)	40(16.5)
	Flap surgery	54(55.1)	8(33.3)	30(55.6)	151(62.4)
	Bone graft	68(69.4)	16(66.7)	48(88.9)	194(80.2)
	Implant	68(69.4)	20(83.3)	40(74.1)	184(76.0)
Do you believe that antibiotic resistance is of growing concern?	No	0(0.0)	0(0.0)	0(0.0)	5(2.5)
	Yes	98(100.0)	24(100.0)	54(100.0)	237(97.9)
First-choice antibiotic prescribed to patients allergic to Pencillin	Spirameyin + Metronidazole	6(6.1)	0(0.0)	2(3.7)	14(5.8)
	Clindamycin	32(32.6)	4(16.7)	8(14.8)	78(32.2)
	Clarithromycin	12(12.2)	4(16.7)	6(11.1)	18(7.4)
	Azithromycin	46(46.9)	14(58.3)	32(59.3)	118(48.8)
	Others (Specify)	2(2.0)	2(8.3)	6(11.1)	14(5.8)
First choice of antibiotic for a pregnant patient	Spiramycin	0(0.0)	0(0.0)	0(0.0)	2(0.8)
	Amoxicillin + Clavulantic acid	6(6.1)	10(18.5)	2(8.3)	24(9.9)
	Amoxicillin	58(59.2)	28(51.9)	8(33.3)	130(53.7)
	Refer to gynaecologist	34(34.7)	16(29.6)	14(58.3)	86(35.5)

* Both practice type and factors had multiple responses; thus, the number would be greater than total numbers of study population.

**Table 4:** Univariate and multivariate logistic regression to find factors associated with antibiotic regimen perceived as most effective by respondents.

Variable	Category	Amoxicillin + Metronidazole [n = 124]	Others [n = 204]	Odds Ratio for Selection of Amoxicillin + Metronidazole as the Regimen Perceived as Most Effective [95% CI]	*p*-Value	Adjusted Odds R atio [95% CI]	*p*-Value
Gender	Male	38(30.6)	68(33.3)	0.88[0.55 to 1.43]	0.613		
	Female	86(69.4)	136(66.7)	1.0 (ref)			
Clinical experience	<5	68(54.8)	97(47.5)	1.54[0.69 to 3.46]	0.294	1.33[0.57 to 3.13]	0.513
	5–9	30(24.2)	61(29.9)	1.08[0.46 to 2.57]	0.858	0.81[0.32 to 2.0]	0.641
	10–14	16(12.9)	24(11.8)	1.47[0.55 to 3.91]	0.443	1.29[0.46 to 3.59]	0.630
	>=15	10(8.1)	22(10.8)	1.0 (ref)		1 (ref)	
Academic Institution	Present	28(22.6)	70(34.3)	0.56[0.34 to 0.93]	0.023	0.65[0.37 to 1.13]	0.130
	Absent	96(77.4)	134(65.7)	1.0 (ref)		1(ref)	
Private practice	Present	98(79.0)	144(70.6)	1.57[0.93 to 2.66]	0.088	1.45[0.80 to 2.61]	0.217
	Absent	26(21.0)	60(29.4)	1.0 (ref)		1.0 (ref)	
Hospital Based	Present	20(16.1)	34(16.7)	0.96[0.53 to 1.76]	1.00		
	Absent	104(83.9)	170(83.3)	1.0 (ref)		-	
Health care	Present	8(6.5)	16(7.8)	0.81[0.34 to 1.95]	0.636	-	
	Others	116(93.5)	188(92.2)	1.0 (ref)		-	
Bone graft	Yes	102(82.3)	154(75.5)	1.51[0.86 to 2.64]	0.147	1.58[0.88 to 2.84]	0.128
Abscess Drainage	Yes	96(77.4)	157(77.0)	1.03[0.60 to 1.75]	0.924	-	-
Flap Surgery	Yes	78(62.9)	115(56.4)	1.31[0.83 to 2.07]	0.243	1.59[0.96 to 2.64]	0.074
Implants	Yes	94(75.8)	148(72.5)	1.19[0.71 to 1.98]	0.514		
Crowning lengthening	Yes	12(9.7)	34(16.7)	0.54[0.27 to 1.08]	0.071	0.47[0.18 to 1.19]	0.112
Gingivectomy	Yes	10(8.1)	30(14.7)	0.51[0.24 to 1.08]	0.067	0.56[0.21 to 1.48]	0.242
Frenectomy	Yes	16(12.9)	24(11.8)	1.11[0.57 to 2.19]	0.761	-	
Scaling	Yes	4(3.2)	2(1.0)	3.37[0.61 to 18.57]	0.149	-	

## Data Availability

The original contributions presented in this study are included in the article/Supplementary Materials. Further inquiries can be directed to the corresponding authors.

## Supplementary Materials

The following supporting information can be downloaded at: https://www.mdpi.com/article/10.3390/healthcare14193226/s1.
