# Supplementary material for: Antibiotic Prescribing Patterns and Antimicrobial Stewardship Among Indian Periodontists: A Cross-Sectional Survey
Source: Healthcare (Basel). 2026 Oct 1;14(19):3226. doi: 10.3390/healthcare14193226 (PMC13665554; doi:10.3390/healthcare14193226)
Supplement: Supplementary file 1 [file healthcare-14-03226-s001.zip › healthcare-4517020-supplementary.pdf]

STROBE Statement—

	Item No.	Recommendation	Page No.	Relevant text from manuscript
**Title and abstract**	1	(*a*) Indicate the study's design with a commonly used term in the title or the abstract	1	Antibiotic Prescribing Patterns and Antimicrobial Stewardship Among Indian Periodontists: A Cross-Sectional Survey.- A cross-sectional questionnaire survey was conducted among Indian periodontists (January-April 2024), distributed via institutional email and LinkedIn. (...).
		(*b*) Provide in the abstract an informative and balanced summary of what was done and what was found	1	Of 500 invited, 328 responded (65.6%). Respondents were mostly female (67.7%), mean age 32.25±6.05 years; 50.3% had <5 years' experience and 98.6% held an MDS in Periodontology. Amoxicillin plus metronidazole was the regimen most frequently perceived as effective by respondents (37.8%), followed by amoxicillin-clavulanic acid (35.4%) and amoxicillin alone (15.9%). Oral administration was preferred (89.0%). Prophylactic antibiotics were prescribed for infective endocarditis/CABG indications by 89.6%, while 48.8% did not prescribe after laser-assisted surgery (…)
**Introduction**				
Background/rationale	2	Explain the scientific background and rationale for the investigation being reported	2	” the lack of standardized guidelines for antibiotic prescription in periodontal diseases contributes to considerable variability in prescribing behaviours among practitioners [3]. Despite existing recommendations advocating cautious and evidence-based antibiotic use, India, like many other regions, lacks specific standardized clinical practice guidance addressing antibiotic prescribing in periodontal practice. This may contribute to differences in antibiotic selection and prescribing practices across clinicians and practice settings.<br>Given the global urgency surrounding antimicrobial resistance and the variability in antibiotic prescribing practices, there is a need to characterize antibiotic prescribing practices among Indian periodontists and to identify professional factors associated with such practices. Examining differences according to years of clinical experience and practice setting may provide insight into factors contributing to prescribing variability and help identify areas for targeted antimicrobial stewardship. To date, there is limited evidence describing antibiotic prescribing practices and

				perceptions regarding antimicrobial resistance among periodontists in India" (..)
Objectives	3	State specific objectives, including any prespecified hypotheses	2	"this study aimed to assess antibiotic prescribing patterns and attitudes toward antimicrobial resistance among Indian periodontists, including antibiotic selection, prescribing frequency, routes of administration, and clinical and prophylactic indications. The study further evaluated whether prescribing practices differed according to years of clinical experience and practice setting and identified professional factors independently associated with selection of the antibiotic regimen considered most effective for periodontal therapy"(..)
**Methods**				
Study design	4	Present key elements of study design early in the paper	2,3	The study was designed as a cross-sectional, questionnaire-based survey targeting qualified periodontists in India. (..)<br>The questionnaire consisted of three sections comprising 17 questions, with a combination of single-response and multiple-response items. The first section collected general and professional characteristics, including sociodemographic and educational information. The second section assessed antibiotic prescribing practices, including antibiotic selection and prescribing practices in different clinical and prophylactic situations. This section also included a question assessing which antibiotic regimen respondents considered most effective for periodontal therapy. The third section assessed attitudes and perceptions regarding antimicrobial resistance.(…)
Setting	5	Describe the setting, locations, and relevant dates, including periods of recruitment, exposure, follow-up, and data collection	2,3	The study was conducted between January and April 2024. The study population comprised dental graduates who had completed postgraduate specialization in Periodontology (MDS or an equivalent recognized postgraduate qualification) and were practicing as periodontists in India. BDS graduates without postgraduate specialization in Periodontology were excluded from the study.(…) A follow-up reminder was sent after one week (…)
Participants	6	*Cross-sectional study*—Give the eligibility criteria, and the sources and methods of selection of participants	2,3	The study population comprised dental graduates who had completed postgraduate specialization in Periodontology (MDS or an equivalent recognized postgraduate qualification) and were practicing as periodontists in India. BDS graduates without postgraduate specialization in Periodontology were excluded from the study (…)

Variables	7	Clearly define all outcomes, exposures, predictors, potential confounders, and effect modifiers. Give diagnostic criteria, if applicable	3,4	The factors considered gender, clinical experience, practice type, post-operative condition (…)The questionnaire comprised heterogeneous items covering different domains, including antibiotic prescribing practices, clinical and prophylactic indications, and perceptions regarding antimicrobial resistance. These items were intended to assess distinct aspects of antibiotic prescribing behavior (…)
Data sources/ measurement	8*	For each variable of interest, give sources of data and details of methods of assessment (measurement). Describe comparability of assessment methods if there is more than one group	3,4	The questionnaire comprised heterogeneous items covering different domains, including antibiotic prescribing practices, clinical and prophylactic indications, and perceptions regarding antimicrobial resistance. These items were intended to assess distinct aspects of antibiotic prescribing behavior and perceptions rather than a single underlying construct. Cronbach's alpha was initially 0.35 and was recalculated following refinement of the questionnaire, yielding a coefficient of 0.40. Although the alpha value remained low, it was interpreted in the context of the heterogeneous nature of the questionnaire and the absence of a single underlying construct across all items. Therefore, Cronbach's alpha was not considered an appropriate measure of the internal consistency of the final questionnaire. The questionnaire was retained based on its content relevance, face validity, and comprehensive coverage of the predefined domains of the study (...)
Bias	9	Describe any efforts to address potential sources of bias	3	self-reported prescribing subject to bias, possible selection/non-response bias from recruitment via email and LinkedIn, multiple-response items complicating comparisons, and a low Cronbach's alpha (α=0.35).
Study size	10	Explain how the study size was arrived at	3,4	A total of 328 of the 500 invited periodontists responded to the survey, yielding a response rate of 65.6%. Among the respondents, 134 (40.8%) were aged 25-30 years, 100 (30.5%) were aged 30-35 years, 60 (18.3%) were aged 35-40 years, and 34 (10.4%) were aged ≥40 years. The mean age of the respondents was 32.25 ± 6.05 years (range: 25-69 years). Female respondents constituted 222 (67.7%) of the sample, while 106 (32.3%) were male. The mean age of male respondents was higher than that of female respondents (34.57 ± 7.93 vs. 31.30 ± 4.56 years), with a mean difference of 3.27 years ($p<0.001$).

Continued on next page

Quantitative variables	11	Explain how quantitative variables were handled in the analyses. If applicable, describe which groupings were chosen and why	4	following Bonferroni adjustment for the multiple comparisons, the associations observed for crown lengthening and flap surgery were no longer statistically significant. Other prescribing characteristics did not demonstrate statistically significant associations with clinical experience. Because multiple responses were permitted for practice setting, factors influencing brand selection, and postoperative conditions, these variables were analyzed descriptively where appropriate and percentages were not expected to sum to 100% as depicted in Table 2. (…)
Statistical methods	12	(*a*) Describe all statistical methods, including those used to control for confounding	4	The collected responses were analyzed using IBM SPSS Statistics version 23.0 (IBM Corp., Chicago, IL, USA). Descriptive statistics were used to summarize responders' characteristics and questionnaire responses and data presented as frequencies and percentages. Chi-square or Fisher's exact tests were performed to find associations between questionnaire response variables and years of clinical experience.(..)
		(*b*) Describe any methods used to examine subgroups and interactions	4	The antibiotics reported by respondents were classified according to the World Health Organization (WHO) AWaRe (Access, Watch and Reserve) classification. The WHO AWaRe classification was used to categorize the individual antibiotics reported in response to the questionnaire item assessing the most prescribed antibiotic. Each respondent contributed one response to this item
		(*c*) Explain how missing data were addressed	-	-
		Cross-sectional study—If applicable, describe analytical methods taking account of sampling strategy	5	These items were intended to assess distinct aspects of antibiotic prescribing behavior and perceptions rather than a single underlying construct. Cronbach's alpha (…)
**Results**				
Participants	13*	(a) Report numbers of individuals at each stage of study—eg numbers potentially eligible, examined for eligibility, confirmed eligible, included in the study, completing follow-up, and analysed	5,6	Table 1. Descriptive statistics of the responders<br>* Total percentage is more than 100 due multiple response
Descriptive data	14*	(a) Give characteristics of study participants (eg demographic, clinical, social) and information on exposures and potential confounders	7	Table 2. Association of experience in clinical practice (Number of years) with questionnaire.<br>* Multiple responses were permitted for these items; therefore, category totals may exceed 328 and percentages may not sum to 100%. Each category was analysed separately; # Fisher's exact test, $ collapsed in the other categories for finding association.
		(b) Indicate number of participants with missing data for each variable of interest	7	No missing data were present in the analyses
Outcome data	15*			

		Cross-sectional study—Report numbers of outcome events or summary measures	8-9	Table 3. The table shows the association between practice type and questionnaire.<br>* Both practice type and factors had multiple responses , thus the number would be greater than total numbers of study population.
Main results	16	(*a*) Give unadjusted estimates and, if applicable, confounder-adjusted estimates and their precision (eg, 95% confidence interval). Make clear which confounders were adjusted for and why they were included	8	Unadjusted and adjusted odds ratios (ORs) with corresponding 95% confidence intervals (CIs) were reported.

Continued on next page

Other analyses	17	Report other analyses done—eg analyses of subgroups and interactions, and sensitivity analyses	8,9	A statistically significant association was observed between clinical experience and the frequency of antibiotic prescription for periodontal infections (p=0.024). Clinical experience was also significantly associated with antibiotic prescription following laser-assisted periodontal surgery (p=0.048), as well as antibiotic prescription following crown lengthening (p=0.008) and flap surgery (p=0.009).
**Discussion**				
Key results	18	Summarise key results with reference to study objectives	10,11	The present study identified substantial variation in antibiotic prescribing among Indian periodontists by clinical experience and practice setting. Academic institutional practice was associated with selecting amoxicillin plus metronidazole univariably, but not after multivariable adjustment.(…)
Limitations	19	Discuss limitations of the study, taking into account sources of potential bias or imprecision. Discuss both direction and magnitude of any potential bias	11	Limitations include the cross-sectional design, self-reported prescribing subject to bias, possible selection/non-response bias from recruitment via email and LinkedIn, multiple-response items complicating comparisons, and a low Cronbach's alpha (α=0.35).
Interpretation	20	Give a cautious overall interpretation of results considering objectives, limitations, multiplicity of analyses, results from similar studies, and other relevant evidence	12,13	Given that high awareness coexisted with measurable prescribing variability in this survey, isolated awareness campaigns are unlikely to suffice; stewardship must combine standardized protocols, targeted education, and institutional surveillance to close the gap between recognizing resistance as a concern and consistently achieving AWaRe-concordant prescribing.
**Other information**				
Funding	22	Give the source of funding and the role of the funders for the present study and, if applicable, for the original study on which the present article is based	13	Funding: This research received no external funding.

*Give information separately for cases and controls in case-control studies and, if applicable, for exposed and unexposed groups in cohort and cross-sectional studies.

Note: An Explanation and Elaboration article discusses each checklist item and gives methodological background and published examples of transparent reporting. The STROBE checklist is best used in conjunction with this article (freely available on the Web sites of PLoS Medicine at http://www.plosmedicine.org/, Annals of Internal Medicine at http://www.annals.org/, and Epidemiology at http://www.epidem.com/). Information on the STROBE Initiative is available at www.strobe-statement.org.
